# Behavioral and Emotional Changes One Year after the First Lockdown Induced by COVID-19 in a French Adult Population

**DOI:** 10.3390/healthcare10061042

**Published:** 2022-06-03

**Authors:** Sylvie Rousset, Aurélie Level, Florine François, Laurent Muller

**Affiliations:** 1 University Clermont Auvergne, UNH, UMR1019, INRAE, 63000 Clermont-Ferrand, France; ffrancois@chu-clermontferrand.fr; 2 University Grenoble Alpes, CNRS, GAEL, INRAE, Grenoble INP, 38000 Grenoble, France; aurelie.level@inrae.fr (A.L.); laurent.muller@inrae.fr (L.M.)

**Keywords:** post-lockdown, sedentary behavior, physical activity, food choice, positive emotions, desire to eat, adult, COVID-19, smartphone

## Abstract

(1) Background: The lockdown had various consequences on physical activity and food consumption behaviors. The post-lockdown has been much less studied. The aim of this study is to compare behaviors one year after the first lockdown in a group of normal-weight (NW) or overweight French adults (OW). (2) Methods: Over a period of 4 days, both at the beginning of May 2020 (lockdown) and in June 2021 (free living post-lockdown), the same French adults used the WellBeNet smartphone application to record their sedentary behavior, physical activity (PA), food consumption and emotions. (3) Results: One year post first lockdown, the weight and body mass index increased (+1.1 kg; +0.4 kg.m^−2^, *p* < 0.01), and sedentary behaviors increased (+5.5%, *p* < 0.01) to the detriment of light-intensity activities (−3.3%, *p* = 0.10) in the whole group. Some food categories, such as alcohol, tended to be consumed more (+0.15 portion/day, *p* = 0.09), while fatty, salty and sugary products decreased (−0.25 portion/d, *p* = 0.02) but without a change in the food balance score. A higher number of both positive and negative emotions were scored per day (+9.5, *p* < 0.0001; +2.9, *p* = 0.03), and the positive ones were perceived stronger (+0.23, *p* = 0.09). Simultaneously, the desire to eat was lower (−11.6/100, *p* < 0.0001), and the desire to move remained constant. Sedentary/active behaviors and the desire to eat changed differently in NW and OW adults after the lockdown. (4) Conclusions: In general, the post-lockdown period was less favorable for physical activity practice and resulted in a similar food balance score but was more conducive to mental wellbeing.

## 1. Introduction

The COVID-19 epidemic has forced many governments in the world to confine the population. In France, the first lockdown began in March 2020 and ended mid-May, with a travel ban and the closure of nonessential stores in order to limit human contact and, thus, the spread of the virus [1]. Outdoor activity was limited to a maximum of one hour and to within a radius of one kilometer around the home. In a CREDOC (Centre de Recherche pour l’Étude et l’Observation des Conditions de Vie (https://www.credoc.fr, accessed on 1 July 2021)) survey, 72% of respondents said they were worried about the risks of serious illness, and 63% said they were worried about COVID-19. This atmosphere of threat to health instills a feeling of insecurity on a daily basis [2]. The lockdown had an impact on lifestyle habits such as the physical activity (PA) level and eating behavior. Disease outbreaks often influence and change health-related behaviors.

Many studies in 2020 compared PA behaviors before and during the lockdown and showed contrasting results. A qualitative Canadian study showed that during the COVID-19 pandemic, people reacted differently: some of them maintained PA, others reduced PA due to a lack of time or motivation and others reported they had more time to exercise [3]. In a New Zealand study, moderately pre-lockdown active individuals were significantly more active during and after the lockdown [4]. The reasons given to increase PA were to get outside during the lockdown by engaging in simple activities, such as running or cycling and to maintain physical and psychological wellbeing post-lockdown. In contrast, the pre-lockdown very active participants were less active during and after the lockdown. Their level of PA decreased because they were unable to practice their preferred PA under restrictions imposed by the lockdown, and they may lose their routine habits or develop other habits following changes in their life situation post-lockdown [4]. In Spain, a great reduction in physical activity was observed during the lockdown. It was the European country with the highest rate of inactivity because there was no space at home to carry out exercises [5]. However, post-lockdown, most of the subjects exercised two or three days a week [6].

It has been shown that quarantine and isolation can have effects not only on the level of PA but also on the consumption of unhealthy food and anxiety [7]. As regards diet, some people chose to eat more snacks and fatty, salty and sugary products to cope with depression and anxiety brought on by the epidemic. Other people chose to cook themselves food and consumed more fruit and vegetables during the lockdown than before. More recent studies investigated the post-lockdown period to determine whether behaviors modified by the lockdown living conditions were maintained. In an Australian study, the authors observed a lower quantity of physical activity, poorer mental wellbeing during than after the lockdown; moreover, junk food, soft drinks and alcohol were consumed more during the lockdown [8]. In a Chinese study, the authors compared dietary diversity during and after the lockdown [9,10]. To assess a diversity score, the authors measured the food consumption in 12 categories during both periods and assigned one point for each consumed food category. The mean dietary diversity score was high and similar during and after the lockdown (9.7 ± 2.1 vs. 9.2 ± 2.0). People were more likely to adopt a healthier diet, especially among those who were worried about contracting the virus [11]. People who consumed more raw food, seafood and imported frozen food had higher diversity scores than those whose consumption stayed the same or decreased [9,10]. However, some people adopted irrational behaviors, such as drinking alcohol or vinegar, to prevent COVID-19 during the lockdown, and this practice substantially decreased post-lockdown without disappearing completely. Although the Chinese government had already dismissed this rumor, more than 10% of the studied population was still purposely drinking more alcohol [9,10]. This behavior has also been reported in many other countries. In Italy, the participants reported an increase in healthy food, a decrease in junk food consumption and more time to cook during the lockdown [12]. These changes were stronger for young and restrained eaters. However, these new habits were partially discontinued post-lockdown; the participants consumed less healthy food and cooked less, but the reduction in junk food was maintained. In Spain, cooking at home is a usual habit, and most of the subjects did not order food at home in the post-confinement period. The consumptions of fruit and vegetables increased by 27% and 21%, respectively, in Spanish consumers during the lockdown compared to the pre-COVID-19 period [13] and stayed in an appropriate quantity after the lockdown. Depending on the country, the lockdown drastically changed life conditions, behavioral components and psychological states.

Our goal is to contribute to the literature that examines the impact of lockdowns on health. In particular, we examined whether exiting a period of lockdown improves an individual’s eating habits, physical activity level and psychological states. Accordingly, we assessed dietary intake, physical activities and emotions of a group of French adults during and one year after the first lockdown in the spring of 2020.

In this context of craze for health applications, we chose to use a research mobile app to record behaviors and emotions. This innovative method to collect data is easy, accurate and immediate. First, the intensity of physical activities were recorded automatically by the native accelerometers of the mobile allowing non subjective and precise data. Second, the data were recorded in vivo. Respondents recorded their food consumption and emotions as they experienced them. This avoided the possible information loss that happens when questionnaires are filled out after the event.

## 2. Materials and Methods

### 2.1. Experimental Design

The volunteers downloaded the WellBeNet app at the Play Store (WellBeNet is not applicable to the iOS system. Even if Android represents approximately 80% of the market share, not taking iPhones into account could generate a sampling bias) on their own smartphone and followed the instructions directly from the app for three to five consecutive days, from 11 April to 7 May 2020, and after the lockdown, from 1 June to 3 July 2021. This observational study was conducted according to the guidelines of the Declaration of Helsinki. The protocol was approved by the French Committee for the Protection of Human Subjects (Sud-Est VI). It was registered under the references 2020/CE 19. All participants provided informed consent prior to participating in the study (https://activcollector.clermont.inra.fr/home/publications/InformationConsentGael, accessed on 1 April 2020).

### 2.2. Measures

After entering age, gender, height and weight into the app, volunteers were then asked to use the three WellBeNet features: eMouve, NutriQuantic and EmoSens.

eMouve provides an accurate estimation of time spent in four levels of activity: immobility, light, moderate, and vigorous intensity in the normal weight and overweight volunteers (NW and OW). These activity thresholds were determined in several previous publications [14,15]. The average absolute error of PA intensity estimation was approximately 3.25% compared to the reference methods [14,15]. Volunteers were asked to wear the smartphone in their pant pocket to collect accelerometry data during the waking period (8:00 AM to 10:00 PM). Time spent in each activity was expressed as a percentage of the whole recording time.

NutriQuantic collects the daily number of meals and portions consumed in each of the 11 food (the number of hot drinks was counted but was not associated with a nutritional score) groups divided in four meta-categories: plant products (fruit, vegetables, legumes and nuts), animal products (meat, fish, eggs and dairy products), junk food (fatty, salty and sugary products; snacks and alcohol) and starchy food (refined starchy and whole-grain starchy products). The content of each food category and the size of the portions are presented in the WellBeNet app and in a guide sent to each participant. A food balance score was calculated for each of the 11 food groups according to the number of portions, the French and international nutritional guidelines [16] and international recommendations [17]. The score in each food group varied among 0 (unsatisfactory), 0.5 (intermediate) and 1 (satisfactory). The nutritional balance score of the diet resulted from the sum of the scores obtained by the 11 food groups [18].

With EmoSens, the volunteers assessed their body image, physiological state (desire to eat and to move) and emotions in the morning, at midday and in the evening [19]. To do this, they selected one of the nine silhouettes defined by Stunkard et al. [20] and chose to color it in one of the following hues: orange, yellow, red, gray or white. The orange color is associated with fear and anxiety; red with desire, hate and passion; yellow with happiness, joy and love; gray with depression and sadness; and white with neutrality [21,22]. They then rated on 10-point scales either none, one or more of the 20 emotional terms from the Geneva Wheel [23,24]. Finally, volunteers scored their desire to eat and move on unstructured scales. The scores varied between 0 and 100. To evaluate the relative part of the desire to eat, the score of this one was divided by the sum of the two desires.

### 2.3. Sample

This observational study was conducted with 91 adult volunteers living in Grenoble and the surrounding area. A total of 72% were women, aged 38 years (±8 y), with no difference in age or weight status between the two sexes (Table 1). They were recruited by e-mail by the Grenoble Applied Economics Laboratory (GAEL), which complied with the French General Data Protection Regulation.

During the lockdown, 25% of them were alone; the others were in a family or in a couple. Anthropometric characteristics are shown in Table 1. Height and weight are close to the average for French adults. A total of 63% of the volunteers had a normal body mass index (BMI), between 18.5 and 24.9 kg/m^−2^. A total of 7% were underweight (<18.5 kg·m^−2^), and 30% were overweight or obese (BMI > 25 kg·m^−2^). There were as many overweight people in our sample as in the French population aged 18–39 years (34%, [25]).

### 2.4. Statistical Analyses

The data analysis plan was determined prior to data collection to serve our purpose of comparing behavioral variables during and after the lockdown of normal-weight and overweight volunteers and of volunteers living alone and with family. All variables were tested for normality (Shapiro–Wilk test). For normally distributed variables (immobility and light- and moderate-intensity activities; daily number of meals and portions; whole starch, fruit, nut and dairy products; fatty, salty and sugary products; meat, fish and eggs; desire to eat and to move; and the number and mean of positive emotions and the mean of negative emotions), the differences between periods (lockdown/post-lockdown) were evaluated by a Student’s paired t-tests for all volunteers and for each weight status (normal-weight, NW and overweight, OW). Variations in other non-normally distributed variables were examined using Wilcoxon signed-rank test for paired responses. A MANOVA was also performed on the four variables of immobility, light-, moderate- and vigorous-intensity activity to determine the overall effect of body mass index group (BMI) on the variation in physical activity profile. Statistical analyses were carried out with SAS version 9.4 statistical analysis software.

## 3. Results

Table 2 presents the average changes in all variables considered by weight status. The table includes statistical tests for differences evaluated in the full sample and in NW and OW between the first lockdown period and the following year.

### 3.1. Evolution of Anthropometry

We observed a generalized increase in anthropometric variables. The increase is statistically significant for the full sample of volunteers with an average increase of 1.1 kg in body weight and of 0.49 kg/m² in BMI. This trend is reflected in the NW population (+0.96 kg and 0.32 kg/m^2^) and in the OW population (+1.50 kg and +0.53 kg/m^2^), but it is not statistically significant for the latter.

Regarding family status, the volunteers with families gained weight (+1.2 kg, *p* = 0.04), whereas the increase was nonsignificant for those living alone (+1.0 kg, *p* = 0.13).

### 3.2. Evolution of Physical Activities

**Figure 1:**
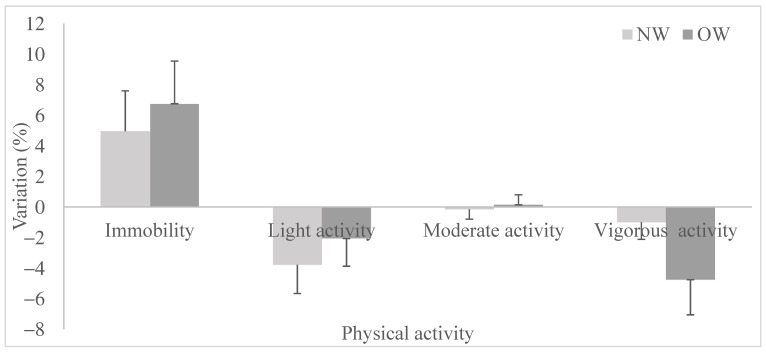
Variation in immobility and PA after lockdown in normal weight and overweight French adults.

Inactivity times increased significantly after the lockdown (+5.5% for the full sample). This increase was mainly at the expense of light activity times, which decreased significantly in the whole sample (−3.3%). However, the pattern of substitutions between activity types differed across the two BMI groups (Manova, F = 2.59, *p* = 0.04). Changes were rather more extreme in OW. First, the shift toward more immobility was more significant in OW (+6.7%) than in NW (+4.9%, Figure 1). Second, immobility times in OW replaced vigorous activity times to a greater extent than light activity times (−4.8% and −2.1%). On the contrary in NW, vigorous and light activity times decreased by −2.1% and −3.3%, respectively, post-lockdown. Finally, the moderate intensity activities remained unchanged for both BMI groups (−0.1%).

Sedentary behaviors increased in volunteers living with families after the lockdown (+6.0%, *p* = 0.001). This was not the case in volunteers living alone (+3.9%, *p* = 0.40).

### 3.3. Evolution of Eating Behaviors

Overall, eating behaviors remained remarkably stable between the two periods. That is, the total number of meals, of servings and the food balance score all remained unchanged (Table 2). Only a few changes in the consumption per food categories were noticeable. One year after the lockdown period, OW consumed more plant products in general (+0.7 servings per day) and fruits in particular (+0.6). NW consumed fewer fatty, salty and sugary products (−2.2) and fewer starchy products (−1.7) but also more alcohol (+0.2).

Vegetable, meat, fish and egg consumption after the lockdown was lower than during the lockdown in volunteers living alone (−0.4 serving/d, *p* = 0.07 and −0.2 serving/day, *p* = 0.07), resulting in a lower food balance score (−1.9, *p* = 0.07). Volunteers with families consumed fewer fatty, salty and sugary products after the lockdown period (−0.3 serving/day, *p* = 0.01).

### 3.4. Evolution of Emotions

First, the desire to eat decreased dramatically, especially in NW. On the other hand, the desire to move remained stable, resulting in an improved balance between the desires to eat and to move (Table 2). Second, participants were happier one year after the lockdown: (i) the average ratings of positive emotions increased significantly; (ii) silhouettes were more frequently colored in yellow, which is associated with happiness, to the detriment of white (neutral) silhouettes; and (iii) participants reported on average nine more positive emotions than during the lockdown. Nevertheless, they also reported three more negative emotions on average and increased the use of red, usually associated with passion but also hate. Third, the corpulence of the chosen silhouettes remained unchanged, except in NW who perceived themselves to be thinner one year after the lockdown.

Emotions perceived by the volunteers living alone were more positive after the lockdown as indicated by the increasing use of the color yellow (+18%, *p* = 0.06), the higher number and mean of positive emotions (+9, *p* = 0.006; +0.7, *p* = 0.04) and the lower mean of negative emotions (−0.6, *p* = 0.04). In volunteers living with families, both the positive and negative emotions were scored at a higher number (+10, *p* < 0.0001; +4, *p* = 0.01, respectively) but with no difference in the mean. The red color was also more used (+4%, *p* = 0.001). In both subgroups, the desire to eat was lower (−15.1, *p* = 0.03; −10.4, *p* = 0.002) after the lockdown period.

## 4. Discussion

Our results contradict those obtained in Spain and Australia that show less sedentary and more active behavior after the lockdown [6,8]. Travel and movement restrictions differed from one country to the next. In France, people could engage in outdoor physical activities for one hour a day, walk the dog and go shopping to the grocery store/supermarket. In Spain, on the other hand, only essential shopping was authorized during the lockdown. Outdoor physical activities were therefore made impossible, and the lack of suitable space at home led to sedentary behaviors [5]. The Spaniards seized the lifting of the lockdown as an opportunity to resume exercising [6]. Furthermore, the end of the first lockdown in France coincided with the return to work where telecommuting was strongly favored. Thus, in August 2021, 59% of French people stayed at home and telecommuted for two to four days a week, but with less free time to move and more time sitting in front of a screen [26].

How physical activity is measured may also explain the differences among studies. While the current literature almost exclusively uses questionnaires, physical activity here was derived from accelerometry data that accurately discriminate four activity categories to rank intensity level [14,15]. For instance, questionnaires cannot evaluate activities of light-intensity or short duration; they collect data that are subjective and approximate. This enabled us to distinguish between NW participants who primarily decreased low-intensity activities and overweight participants who instead rather decreased periods of vigorous activity. Other studies reporting objective measures of physical activities are scarce. Examples include obese adolescents in Maltoni et al. [27] and patients undergoing bariatric surgery in Andreu et al. [28].

Regarding food intake, the literature provides mixed results. While some studies report healthier eating habits (more fruit and vegetables and less junk food consumption) due in part to greater involvement in meal preparation during the lockdown [29,30], others report increased consumption of unhealthy food such as frozen pizza, cheese, sausage and potato chips in Deschasaux-Tanguy et al. and Nilsen [31,32] and snacks, cereals and sweets in Pellegrini et al. [33]. Our results are more in line with the latter observation, with greater consumption of fatty, sugary, salty and starchy food for NW and fewer plant products for OW during than after the lockdown. Nevertheless, the extent of these changes is not sufficient to generate a significant improvement in the nutritional balance score after the lockdown. A significant portion of our sample continued to telecommute part-time (49.3%) or full-time (29.6%) even after the lockdown. By staying home, they may have retained their lockdown eating habits.

In contrast to results from other countries [9,10,34], greater alcohol intake was observed post-lockdown. Alcohol consumption is an essential part of French culture and is associated with social interactions. In Guignard et al., one in five French drinkers reported lower consumption, and only one in ten reported higher consumption during the lockdown [35]. Restrictions placed on social interactions during the lockdown reduced opportunities to drink with acquaintances. These restrictions ceased with the reopening of bars and restaurants, and, thus, there were chances to share convivial moments with others and with alcohol.

In line with the literature, participants reported more of a desire to eat during the lockdown period than after. Gao et al. (2021) associated the increased desire of Chinese respondents to eat high calorie food during the lockdown with social media exposure [36]. Sanchez et al. (2021) identified anxiety as the top factor explaining stronger and more frequent hunger sensations during than before the lockdown for 74% of their Spanish respondents [37]. Previous studies showed that emotional eating may be associated with stressful life events [38] and anxiety [39]. Our French respondents instead appeared more emotional after the lockdown; they reported significantly more emotions, both positive and negative, and they reported significantly fewer neutral feelings. Moreover, they did not appear significantly less anxious after the lockdown. Therefore, as in the Andreu et al. (2022) study on obese patients [28], we found no evidence of more emotional eating during than after the lockdown.

Finally, we found that our participants gained weight between the two periods. Our anthropometric measurements were consistent with those showed by Zeigler [40]. Our data on emotions, nutrition and physical activity only partially explained this weight gain. First, anxiety, loneliness and boredom could lead to the consumption of palatable food and finally to weight gain [41]. As we have just seen, we did not find a decrease in these feelings after the lockdown. Nevertheless, this nondecrease did not imply a nonexistence. It could simply mean that the end of the lockdown did not make these emotions disappear, thus reviving the hypothesis of emotional eating, both during and after the lockdown. Second, respondents showed more desire to eat and consume larger quantities of starchy, fatty, salty and sugary products during the lockdown. However, these changes were not large enough to modify the food balance score. Finally, respondents were more active during the lockdown than after the lockdown, which might at first glance be at odds with the post-lockdown weight gain. Our second weight measurement occurred one year after the first lockdown. In the meantime, participants returned to work primarily through telecommuting, and two more additional lockdowns took place. Thus, the French continued to spend most of their time at home, preserving the emotional eating of the lockdown but with less free time to exercise.

Some differences in behaviors and emotions were noticeable between volunteers living alone and those living with families. The change in eating behavior during the post-lockdown period was detrimental to the health of those living alone (lower food balance score). The increase in post-lockdown sedentary behavior was detrimental in people with families. With regard to emotions, the post-lockdown period improved the condition of people living alone. Deprived of social relations during the lockdown, they may have suffered from a lack of contact. Social relationships can alleviate distress and anxiety [42]. They were associated with lower stress levels, lower worry about COVID-19 and less fatigue [43]. These findings show that social connections play a significant role in resilience by mitigating negative physical and mental health outcomes. This change in positive emotion between the two periods was less pronounced for those living with family. There was a higher number of negative emotions and a larger use of the color red. The post-lockdown period and the return-to-work could be a source of stress and worsening of the evening mood with more constrained schedules, such as an earlier and shorter sleep [44].

This work has several limitations. First, the results are contingent on a specific sample. Only volunteers who owned an Android smartphone were eligible to participate. Those who had an iPhone were not selected. Fortunately, they are few in number: only 20% of people had an iPhone. Another bias relates to the location of our sample and the unbalanced gender size. Our sample included more women exclusively from the Grenoble area. Second, the results may be subjected to a reporting bias. Whereas physical activities are automatically recorded by the WellBeNet application, diets and emotions were self-reported. This could lead to oversights, misperceptions and, more generally to a reporting bias. To our knowledge, there are currently no measures based on actual observation of diets and emotions under such ecological conditions. Moreover, examining differences rather than absolute levels mitigates such reporting problems.

The strength of the study was the use of a smartphone app designed for and developed by the Human Nutrition Research team of INRAE. Time spent in sedentary behavior and physical activity was accurately measured, and the food servings could be recorded immediately after meals. The emotions could also be recorded in real time. All of this data were recorded into the same app. As the volunteers used their own smartphone, there was no risk of contagion between the volunteers and researchers.

## 5. Conclusions

This study investigated the effects of leaving the lockdown. The WellBeNet smartphone app provided data on anthropometric measurements, food intake, physical activities and perceived emotions of 91 adults during and one year after the French first lockdown. Body weight and BMI increased between the two periods. Participants were more sedentary and engaged in less light-intensity activity after the lockdown. The composition of diets changed little with fewer fatty, salty and sugary products and less starchy food but more alcohol in the post-lockdown period. Overweight participants also consumed more fruit. The desire to eat was less intense than during the lockdown. Finally, participants perceived a higher number of emotions, both positive (to a larger extent) and negative (to a lesser extent).

This work contributes to anticipating the negative aspects of a lockdown through preventive actions during a new or upcoming COVID-19 epidemic. Prevention action should address overeating as a reaction to stress or boredom. The population should be educated about the dangers of increased consumption of fatty, salty and sugary food and a decreased consumption of fruit. These unhealthy variations during the lockdowns seem to be unconscious or estimated to be without health damage. Finally, people living alone during a lockdown should receive extra support as they appear to be at greater emotional risk.

## Figures and Tables

**Table 1:** Anthropometric characteristics by gender and weight status (Mean (SD)) at the beginning of the study.

Subsample	*n*	Age (y)	Height (cm)	Weight (kg)	BMI (kg/m^2^)
Women	66	38.3 (7.9)	164.6 (6.5)	66.1 (14.0)	24.5 (5.5)
Men	25	37.6 (8.9)	177.8 (7.3)	77.2 (18.0)	24.4 (5.9)
Normal-weight	64	38.2 (8.5)	168.5 (9.4)	61.7 (8.8)	21.6 (1.8)
Overweight	27	37.9 (7.4)	167.0 (8.6)	86.3 (17.0)	31.3 (6.2)

**Table 2:** Variations after the lockdown in anthropometry, physical activity, food eating and feelings in the full sample and by weight status. *S* is the value of the Wilcoxon signed-rank test; *t* is value of the paired Student’s *t*-test, and *p* is the *p*-value. Means in bold are statistically significant at the 10% level.

	Full Sample			Normal-Weight			Overweight		
	Mean	*S* or *t*	*p*	Mean	*S or t*	*p*	Mean	*S or t*	*p*
**Anthropometry**									
Weight (kg)	**1.10**	S = 300	<0.01	**0.96**	S = 124	0.03	**1.50**	S = 37.5	0.10
BMI (kg/m²)	**0.39**	S = 300	<0.01	**0.32**	S = 126	0.03	0.53	S = 35.0	0.13
**Physical activity**									
*Percent*									
Immobility	**5.49**	t = 2.71	0.01	**4.95**	t = 1.87	0.06	**6.75**	t = 2.41	0.02
Light activity	**−** **3.27**	t = −2.28	0.02	**−** **3.78**	t = −1.99	0.05	−2.06	t = −1.14	0.27
Moderate activity	−0.07	t = −0.14	0.88	−0.15	t = −0.25	0.80	0.14	t = 0.21	0.83
Vigorous activity	−2.13	S = 13.5	0.92	−1.00	S = 57	0.46	−4.77	S = −16	0.50
**Food behavior**									
*Number per day*									
Meal	−0.06	t = −1.50	0.13	−0.12	t = −1.51	0.13	0.07	t = 0.50	0.61
All servings	−0.43	t = −1.31	0.19	−0.75	t = −1.31	0.19	0.23	t = 0.29	0.77
*Serving per day*									
Fruit	0.08	t = 0.64	0.52	−0.18	t = −1.29	0.20	**0.63**	t = 3.95	<0.01
Vegetable	−0.13	t = −1.10	0.27	−0.13	t = −0.92	0.36	−0.14	t = −0.60	0.55
Nut	0.05	t=0.55	0.58	−0.02	t = −0.26	0.79	0.21	t = 1.59	0.12
Legume	−0.03	S=-40	0.64	−0.05	S = −24	0.62	0.01	S = −3.5	0.84
Plant product	−0.04	t = −0.17	0.86	−0.40	t = −1.38	0.17	**0.71**	t = 1.80	0.08
Whole starch	−0.14	t = −0.88	0.32	−0.12	t = −0.55	0.58	−0.20	t = −0.85	0.41
Refined starch	−0.25	t = −1.20	0.23	**−** **0.28**	t = −1.71	0.09	0.10	t = 0.52	0.61
Starch	**−** **0.30**	t = −1.71	0.09	**−** **0.40**	t = −1.87	0.06	−0.09	t = −0.30	0.76
Dairy product	0.00	t = 0.01	0.98	0.15	t = 0.97	0.33	−0.33	t = −1.55	0.13
Meat, fish, eggs	−0.04	t = −0.41	0.68	−0.08	t = −0.73	0.47	0.05	t = 0.32	0.75
Animal product	−0.04	t = −0.21	0.83	0.07	t = 0.33	0.74	−0.27	t = −1.10	0.30
Fatty, salty, sugary	**−** **0.25**	t = −2.20	0.03	**−** **0.28**	t = −1.89	0.06	−0.17	t = −1.10	0.28
Snack	0.01	S = 39.5	0.67	−0.01	S = 14	0.79	0.05	S = 3.00	0.88
Junk food	−0.08	t = −0.50	0.62	−0.08	t = −0.44	0.66	−0.08	t = −0.24	0.81
Alcohol	**0.15**	S = 142	0.09	**0.20**	S = 102	0.05	0.04	S = −0.5	0.99
*Score*									
Food balance	−0.13	t = −0.88	0.38	−0.27	t =−1.40	0.16	0.17	t = 0.79	0.43
**Emotion**									
*Number*									
Positive emotion	**9.50**	t = 6.93	<0.01	**10.00**	t = 5.66	<0.01	**8.54**	t = 3.93	<0.01
Negative emotion	**2.98**	S = 203	0.03	**4.20**	S = 109	0.03	0.63	S = 16.00	0.43
No emotion	−0.04	S = −12	0.15	−0.06	S = −8	0.22	−0.01	t = −0.50	0.99
*Percent*									
Relative desire to eat	**−** **3.69**	t = −2.32	0.02	−3.30	t = −1.56	0.12	**−** **4.42**	t = −1.99	0.06
Orange	−2.00	S = −7.5	0.88	−0.90	S = 10.5	0.72	−4.10	t = −t = 8.50	0.43
Yellow	**7.00**	S = 113	0.04	**6.30**	S = 44.5	0.06	9.20	S = 9.00	0.59
White	**−** **8.00**	S = −139	0.08	**−** **11.00**	S = −67.5	0.08	−3.60	t = −5.50	0.79
Red	**3.00**	S = 106	0.01	**5.30**	S = 53.5	0.02	−0.80	S = 10.00	0.26
Grey	0.00	S = 11	0.67	0.30	S = 11.5	0.39	−0.60	t = −1.50	0.86
*Rating*									
Desire to eat	**−** **11.60**	t = −4.07	0.00	**−14.30**	t = −3.96	0.00	−6.25	t = −1.40	0.17
Desire to move	−1.50	t = −0.69	0.49	−2.40	t = −0.88	0.38	0.27	t = 0.07	0.94
Positive emotion	**0.23**	t = 1.78	0.07	0.20	t = 1.22	0.23	0.28	t = 1.44	0.16
Negative emotion	−0.01	t = −0.06	0.94	0.01	t = 0.05	0.96	−0.05	t = −0.15	0.88
Silhouette	−0.14	S = −141	0.27	**−** **0.30**	S = −137	0.04	0.07	S = 31.00	0.26

## Data Availability

The data presented in this study are available on request from the corresponding author.
